# Nonlinear Effect of Dispersal Rate on Spatial Synchrony of Predator-Prey Cycles

**DOI:** 10.1371/journal.pone.0079527

**Published:** 2013-11-11

**Authors:** Jeremy W. Fox, Geoff Legault, David A. Vasseur, Jodie A. Einarson

**Affiliations:** 1 Department of Biological Sciences, University of Calgary, Calgary, Alberta, Canada; 2 Department of Ecology and Evolutionary Biology, Yale University, New Haven, Connecticut, United States of America; McGill University, Canada

## Abstract

Spatially-separated populations often exhibit positively correlated fluctuations in abundance and other population variables, a phenomenon known as spatial synchrony. Generation and maintenance of synchrony requires forces that rapidly restore synchrony in the face of desynchronizing forces such as demographic and environmental stochasticity. One such force is dispersal, which couples local populations together, thereby synchronizing them. Theory predicts that average spatial synchrony can be a nonlinear function of dispersal rate, but the form of the dispersal rate-synchrony relationship has never been quantified for any system. Theory also predicts that in the presence of demographic and environmental stochasticity, realized levels of synchrony can exhibit high variability around the average, so that ecologically-identical metapopulations might exhibit very different levels of synchrony. We quantified the dispersal rate-synchrony relationship using a model system of protist predator-prey cycles in pairs of laboratory microcosms linked by different rates of dispersal. Paired predator-prey cycles initially were anti-synchronous, and were subject to demographic stochasticity and spatially-uncorrelated temperature fluctuations, challenging the ability of dispersal to rapidly synchronize them. Mean synchrony of prey cycles was a nonlinear, saturating function of dispersal rate. Even extremely low rates of dispersal (<0.4% per prey generation) were capable of rapidly bringing initially anti-synchronous cycles into synchrony. Consistent with theory, ecologically-identical replicates exhibited very different levels of prey synchrony, especially at low to intermediate dispersal rates. Our results suggest that even the very low rates of dispersal observed in many natural systems are sufficient to generate and maintain synchrony of cyclic population dynamics, at least when environments are not too spatially heterogeneous.

## Introduction

Spatially-separated populations in nature often exhibit correlated fluctuations in abundance, population growth rate, and other properties [1]. Even populations separated by hundreds or thousands of kilometers can exhibit spatially-synchronized fluctuations. Although climate synchronization (the Moran effect) can account for some of this phenomenon [2], it cannot account for all of it [3], [4], [5], [6], [7], [8]. Dispersal is a key mechanism generating spatial synchrony in populations experiencing similar density-dependent processes [4], [5], [9], [10]. Dispersal partially mixes spatially-separated populations and couples them together, thereby synchronizing them.

In nature, dispersal rates often are very low, especially at longer distances; most dispersing individuals or propagules don't move very far (reviewed in [11]). It is unclear how low dispersal rates can be while still producing appreciable spatial synchrony. Comparative work suggests that species dispersing longer distances are synchronized over longer distances [3], [12]. This suggests that observed cases of long-distance synchrony represent situations in which even long-distance dispersal rates are high (e.g., [13]). However, theory predicts that the dispersal rate-synchrony relationship can take on various forms, depending on biological details (reviewed in [14]). The spatial scale of synchrony need not align with the spatial scale of the typical dispersal distance [15]. Dispersal rates also can vary over time and space, including due to human activities. For instance, anthropogenic habitat fragmentation can restrict animal movement; conversely, air travel can spread human disease outbreaks around the world in days. Only a few controlled experiments manipulate dispersal rate and examine the effect on spatial synchrony, and these studies consider only one or two non-zero dispersal rates [15], [16], [17], [18]. Ecology currently lacks a quantitative understanding of the expected relationship between dispersal rate and spatial synchrony. For instance, how low can dispersal rates be while still producing appreciable spatial synchrony?

**Figure 1:**
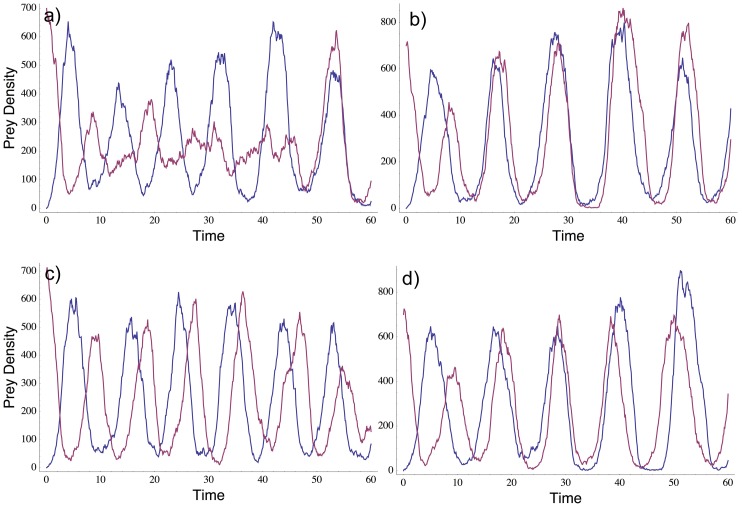
Variation in realized synchrony due to demographic stochasticity. Simulated prey population dynamics in a two-patch Rosenzweig-MacArthur predator-model with demographic stochasticity [33]. In each panel, red and blue lines show prey dynamics in two patches linked by dispersal of prey and predators at the same per-capita rate. The four panels show four different realizations of the model, using the same parameter values and starting from the same, initially-antisynchronous state. Because the model is stochastic, different realizations can have very different behavior, including (a) slow achievement of synchrony (after ∼50 time units in this example), (b) rapid achievement of synchrony (after ∼12 time units) which is subsequently maintained, (c) failure to achieve synchrony during the simulated time period, and (d) achievement of synchrony (after ∼25 time units) which is subsequently lost. Dynamics were simulated using the SSA algorithm of [33], using the following parameter values: attack rate 0.01, handling time 3.0, predator per-capita mortality rate 0.5, predator conversion efficiency 0.4, prey intrinsic rate of increase 2.0 ( =  per-capita birth rate 3.0 - per-capita mortality rate 1.0), prey carrying capacity 1000, prey and predator per-capita dispersal rate 0.05.

Ecology also lacks a complete understanding of the variation around the expected dispersal rate-synchrony relationship. In general, the dynamics of a single realization of any stochastic process can deviate considerably from the average dynamics. For instance, Figure 1 shows prey dynamics in four simulations of a two-patch predator-prey model with density-independent dispersal of both species and demographic stochasticity, and where local dynamics are cyclic. All four simulations began with the same initial conditions, and all four used the same parameter values, yet the realized spatial synchrony of those dynamics varied widely. Fig. 1a shows a slow transition from initially anti-synchronous, anti-phase cycles to synchronous, in-phase cycles, Fig. 1b shows a rapid transition to synchronous, in-phase cycles, Fig. 1c shows cycles that never transition to synchrony over the timeframe shown, and Fig. 1d shows cycles that go into phase and then drift back out of phase. Figure 1 also illustrates variation in cycle period and amplitude between patches within a single simulation, between simulations, and over time. Only extremely high dispersal rates would be expected to produce a reliable, rapid transition to synchronous, in-phase cycles, with little variability among replicate realizations. Spatially-independent environmental stochasticity also would be expected to generate substantial variation in realized spatial synchrony. Variation in the realized level of synchrony also can arise because the probability distribution of synchrony for any given dispersal rate might be multimodal [19]. That is, there might not be one single expected level of synchrony around which realized levels of synchrony vary, but rather several different expected levels, each of which represents an alternative attractor.

We conducted what is to our knowledge the first quantification of the dispersal rate-synchrony relationship for any system by assembling two-patch predator-prey metapopulations in aquatic laboratory microcosms. Each metapopulation comprised a pair of semi-continuous cultures in which the ciliate protist predator *Euplotes patella* fed on the ciliate protist prey *Tetrahymena pyriformis*. Our previous work established this system as a model system for the study of spatial synchrony of predator-prey cycles [15], [18]. Here we subjected replicate metapopulations to one of 11 different rates of dispersal to quantify the dispersal rate-synchrony relationship, and to quantify among-replicate variation in synchrony arising from demographic and environmental stochasticity. This deliberately-simplified microcosm system allowed us to conduct a controlled experiment that would've been impossible to conduct in any natural system. Our results complement observational comparative data from natural systems on the dispersal rate-synchrony relationship (e.g., [3]). Our results provide a baseline aiding the interpretation of future experiments incorporating additional biological complexities.

## Materials and Methods

Experimental units were pairs of microcosms containing the ciliate protist prey species *Tetrahymena pyriformis* and the ciliate protist predator *Euplotes patella*. Culture methods and sampling techniques closely followed previous work [15], [18]. Culture vessels were 100 ml screw-capped glass bottles containing 80 ml of nutrient medium and one wheat seed. Nutrient medium comprised 0.15 g/L of "Protozoan Pellets" (standardized pellets of crushed, dried plant matter; Carolina Biological Supply, Burlington, NC, USA), and one wheat seed per bottle. Bottles were loosely capped to allow gas exchange while preventing contamination. All materials were sterilized (autoclaved) before use.

Forty-eight hours before use, we inoculated the medium with a mixture of unidentified bacteria isolated from the stock cultures of the protists used in the experiment. On day 0 of the experiment, we inoculated all bottles with a small volume of medium from a *Tetrahymena* stock culture, and inoculated one randomly-chosen bottle in each pair with eight *Euplotes* cells drawn from a stock culture of *Euplotes* growing on *Tetrahymena*. We followed the population dynamics (see below for sampling methods) but conducted no dispersal events, until *Euplotes* reached high density and drove *Tetrahymena* to low density. At that point (day 20), we inoculated the other bottle in each pair with eight *Euplotes* cells drawn from the *Euplotes* stock culture. This created pairs of patches with initially anti-phase (maximally out-of-sync) predator-prey cycles. We then began dispersal treatments. The experiment ran until day 92, with our analyses of synchrony considering only the final 72 d, during which dispersal occurred.

We conducted dispersal events daily on weekdays, after sampling. To conduct a dispersal event, bottles were agitated, and a sample of medium and the organisms in it was withdrawn from each bottle and exchanged with the sample from the other bottle in the pair. We used 11 dispersal rate treatments: exchange of 0, 0.125, 0.5, 1, 2.5, 3, 4, 5, 6, 9, or 12.5% of medium per dispersal event. Our previous work used a dispersal rate of 10% of medium per event and three events per week, approximately equivalent to our 6% dispersal rate treatment since we conducted five dispersal events per week in this experiment [15], [18]. Our dispersal rates thus ranged from no dispersal up to dispersal rates substantially higher than those shown to maintain synchrony in previous work [15], [18]. We replicated each dispersal rate treatment five times, for a total of 55 pairs of bottles. This was the largest experiment feasible, given the equipment and personnel available.

For comparability with previous experiments [15], [18], and to provide an additional challenge to the synchronizing effects of dispersal, we subjected all bottles to daily, spatially-asynchronous temperature fluctuations. Temperature fluctuations started at the same time as dispersal; before that, all bottles were maintained in a 20°C incubator. We imposed temperature fluctuations by swapping bottles between two incubators, set at 20°C and 30°C respectively. Each bottle experienced a predetermined sequence of temperatures, generated using the method of [20]. All bottles experienced the same number of days at each temperature, and experienced temperature fluctuations with the same autocorrelation (a 1/*f* ^0.5^ power spectrum). The two bottles in a pair experienced spatially uncorrelated temperature fluctuations (zero cross-correlation). While spatially-uncorrelated temperature fluctuations should tend to inhibit synchronization in all treatments, temperature fluctuations could not create differences in synchrony among treatments because all bottle pairs experienced equally-uncorrelated temperature fluctuations. Each of the five pairs of bottles within a dispersal rate treatment experienced a unique pair of temperature time series, and we used the same five pairs of temperature time series for all dispersal rate treatments. This procedure ensured that our results did not reflect unusual properties of any particular pair of temperature time series, and also ensured that differences among treatments did not reflect differences among the temperature time series used in different treatments.

Once per week beginning on day 7, we agitated the bottles, withdrew 8 ml of medium, and replaced it with 10 ml of fresh sterile medium to prevent exhaustion of the resource base, accumulation of waste products, and to replace medium lost to sampling and evaporation. Medium replacement occurred after sampling and dispersal. Medium replacement did not generate a detectable signal in the population dynamics, and could not create differences among treatments because all bottles experienced the same medium replacement regime.

Bottles were sampled on weekdays starting on day 1, using established methods [18]. Briefly, sampling involved counting the protists in small (*c*. 0.3 ml) samples under a binocular microscope, with dilution and subsampling as necessary to count dense populations. For various technical reasons, it was not possible to automate sampling using a particle counter or image analysis software. Sampling and maintaining the experiment was a full-time job for two people.

### Data processing and statistical analysis

In 13 of the 55 metapopulations, either the prey, the predator, or both went extinct in at least one (usually both) of the two bottles early in the experiment, with the last non-zero density being observed before day 68. Apparent extinctions were confirmed at the end of the experiment by pouring bottle contents into petri dishes and scanning under low magnification. We excluded these metapopulations from the analyses. Extinctions occurred randomly with respect to dispersal rate and the particular temperature sequence experienced, occurred too early in the experiment to be related to realized levels of pre-extinction synchrony, and could not be related to the results in any obvious way. Extinctions likely reflected demographic stochasticity, given that predator-prey cycles in this system are characterized by long periods of low abundance for both prey and predators (see RESULTS). We also excluded from the analyses one metapopulation in which both prey populations were driven to very low but non-zero density immediately after predator addition and remained at very low density for the entire experiment. Prey dynamics in this metapopulation were non-cyclic and dominated by sampling error, so that synchrony could not be meaningfully analyzed or compared to that of the cycling populations.

As in our previous experiment using these culture conditions [15], predator densities were low on average. Sampling error prevents meaningful analysis of predator synchrony.

Our time series were fairly short relative to the period of the predator-prey cycle (>20 d; see RESULTS). Further, inspection of the time series indicated that cycle period varied both over time and among bottles, a feature of the data we discuss below. For this reason, we could not use spectral analysis or wavelets to quantify predator-prey cycle periods or the phase difference between paired cycles as in [15]. Instead we summarized prey synchrony in each metapopulation by transforming prey densities as log(*n*/ml+1), and then calculating the cross-correlation between transformed prey densities in the two bottles in each pair. This approach was used in the previous theoretical and experimental work on which the present study builds [15], [18], [21]. Square root transforming prey densities before calculating cross-correlations led to similar results. We calculated cross-correlations over the entire 72 d following initiation of dispersal, rather than trying to quantify temporal changes in synchrony during this period of time, because cross-correlations calculated over short time windows necessarily are based on only a few data points and so are dominated by sampling error. Linearly detrending the transformed prey densities before calculation of cross-correlation coefficients did not qualitatively affect the results and produced only minor quantitative changes. We applied Fisher's *z* transformation to the cross-correlation coefficients to normalize them before analysis, but note that this made no qualitative difference, as most of the cross-correlations were non-extreme.

We fit several different linear and nonlinear regression models to the relationship between dispersal rate (% medium exchanged per event) and prey synchrony (*z*-transformed cross-correlation) in order to identify the best model. The most complicated continuous model was a flexible, asymmetric sigmoid model *y* = [*ax*/(*b*+*x*)][1+(1-*cx*)e^−*cx*^]. In this model, the parameter *c* governs the location of the inflection point of the sigmoid curve on the *x­*-axis; the inflection point is located at *x* = 1/*c*. This choice of sigmoid function necessarily is somewhat arbitrary, but various other asymmetric sigmoid functions gave similar results (not shown). We included this model among the candidates because recent theory predicts an asymmetrical sigmoid relationship between dispersal rate and spatial synchrony in a system similar to our experimental system [21]. We also considered the simpler saturating model *y* = *ax*/(*b*+*x*), the linear model *y* = *ax*, and the null model *y* = *a*, which lacks any effect of dispersal rate on synchrony. In the limit as *c*→∞, the asymmetrical sigmoid model approaches the saturating model. Finally, we also fit a discontinuous nonlinear model, a piecewise linear regression with a single discontinuity. While there is no biological rationale for a piecewise linear relationship between synchrony and dispersal rate, a piecewise linear function can approximate a relationship with a relatively sharp transition between two phases. Inspection of the residuals for the best-fitting model indicated conformity with statistical assumptions. We compared the models using AIC and (where appropriate) likelihood ratio tests. All statistical analyses were performed in R 2.15.1, with function nls and optimization algorithm "port" used to fit the continuous nonlinear models and function segmented.lm from the segmented package used to fit the discontinuous nonlinear model.

## Results

**Figure 2:**
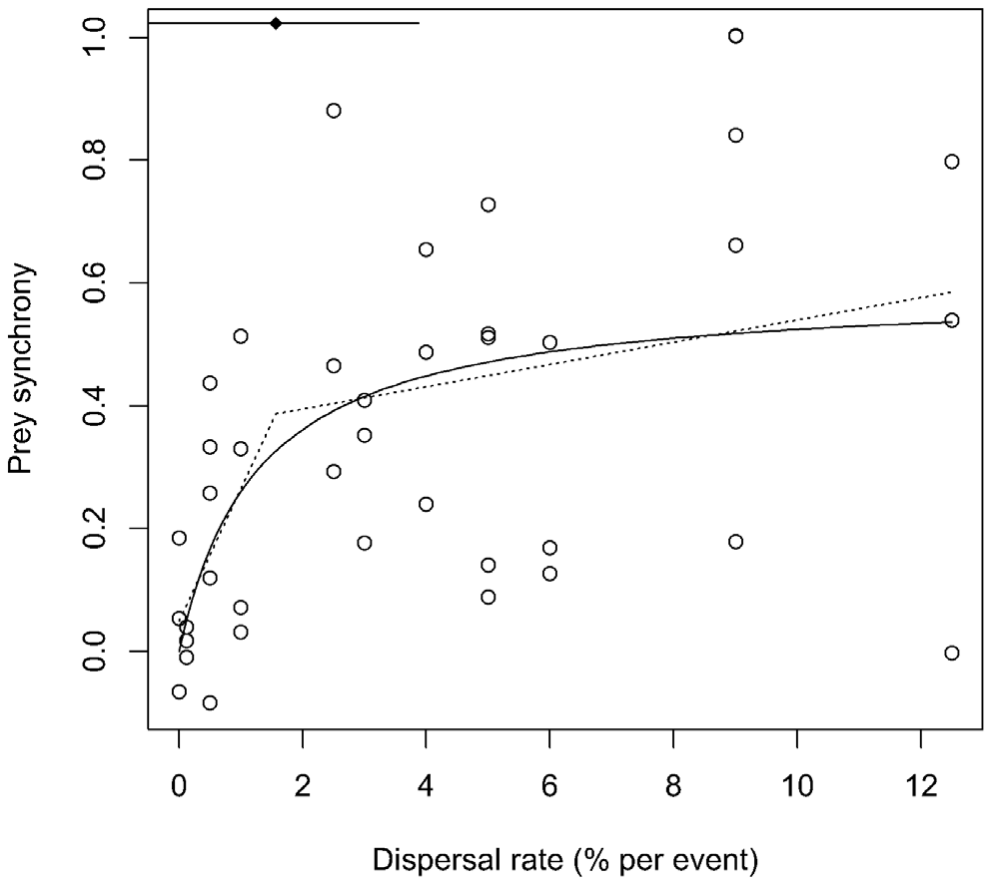
Prey synchrony vs. dispersal rate. Prey synchrony (*z*-transformed cross-correlation of log_10_-transformed prey abundances) as a function of dispersal rate. Each open point gives results from one replicate pair of bottles. The solid curve is *y* = *ax*/(*b*+*x*) with estimated parameters (95% likelihood profile c.i.) of *a* = 0.59 (0.39, 1.25), *b* = 1.27 (0.14,8.50). The curve *y* = [*ax*/(*b*+*x*)][1+(1-*cx*)e^−*cx*^] with estimated parameters (95% likelihood profile c.i.) of *a* = 0.59 (0.45, 1.26), *b* = 1.22 (0.45, 8.62), *c* = 14.66 (14.66, 44.64) is hidden by the solid curve. The dotted curve is a piecewise linear regression. The black diamond indicates the estimated location and 95% confidence interval for the discontinuity of the dotted curve. See [34] for review of the concept of profile confidence intervals.

Prey synchrony was near-zero on average in the absence of dispersal, as intended and confirming that weekly medium replacement was too small and infrequent a perturbation to affect synchrony (Fig. 2). Dispersal rates as low as 0.5% per event were capable of producing levels of synchrony substantially higher than those observed in the no-dispersal treatment (Fig. 2). Dispersal rates as low as 2.5% per event were capable of producing quite high synchrony (cross-correlation >0.7).

The saturating model *y* = *ax*/(*b*+*x*) had the lowest AIC. The saturating model was estimated to be somewhat closer to the unknown true model than the asymmetrical sigmoid model (ΔAIC = 1.9). Further, the maximum likelihood parameter estimates for the asymmetrical sigmoid model produced a curve nearly identical to the saturating model (Fig. 2). The saturating model was estimated to be somewhat closer to the unknown true model than the piecewise linear model (ΔAIC = 3.7), which required more parameters to produce a similar fitted relationship (Fig. 2). The saturating model was estimated to be much closer to the unknown true model than either the linear model or the null model (ΔAIC = 10.8 and 13.4, respectively). The saturating model also fit the data significantly better than the null model *y* = *a* in a likelihood ratio test, indicating that synchrony did vary significantly with dispersal rate (*F* = 17.9, *P*<0.001 with 1 df).

**Figure 3:**
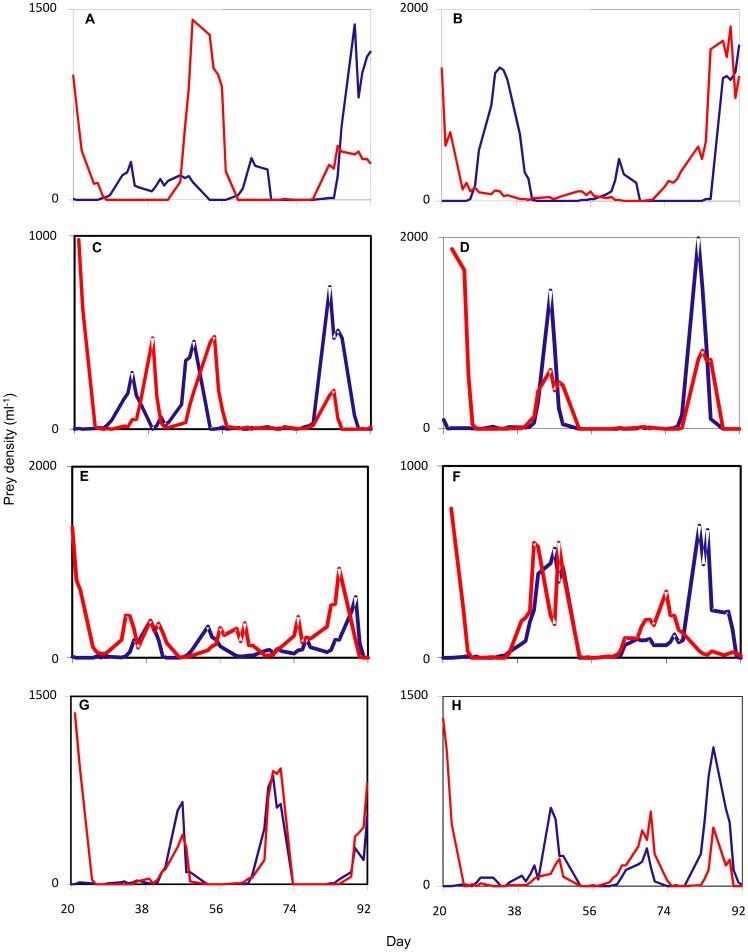
Representative prey population dynamics. Red and blue lines in each panel give prey dynamics in two patches linked by dispersal, starting from day 20 when dispersal was initiated. (a-d) Failure to achieve synchrony with a dispersal rate of 0.125% per event, (c) slow achievement of synchrony with a dispersal rate of 5% per event, (d) rapid achievement of synchrony with a dispersal rate of 5% per event, (e) failure to achieve synchrony with dispersal rate of 2.5% per event, (f) rapid achievement of synchrony which was subsequently lost with dispersal rate of 2.5% per event, (g-h) rapid achievement of synchrony with a dispersal rate of (g) 9% or (h) 12.5% per event. Compare c-f to Figure 1.

While prey synchrony increased on average with increasing dispersal rate, there was substantial variation around this trend (Fig. 2). Replicates with the same dispersal rate often exhibited very different levels of synchrony. Figure 3 illustrates the population dynamics underpinning this variability. As intended, paired bottles invariably were anti-synchronous when dispersal began on day 20, with one prey population at high density and the other at low density. Subsequent dynamics clearly were cyclic in all bottles included in the analysis. However, the realized level of synchrony varied widely. Metapopulations that experienced very low dispersal rates (≤1% per event) rarely went into phase quickly and so never exhibited high prey synchrony (Fig. 3a,b). Conversely, metapopulations that experienced the highest dispersal rates (≥9% per event) typically went into phase quickly and so typically exhibited relatively high prey synchrony (Fig. 3g,h). At dispersal rates in between these extremes, prey cycles sometimes remained out of phase until late in the experiment (Fig. 3c), sometimes went into phase quickly (Fig. 3d), sometimes never went into phase at all (Fig. 3e), and sometimes went into phase quickly only to subsequently drift out of phase (Fig. 3f). This range of behavior closely resembles that produced by a spatial predator-prey model incorporating demographic stochasticity (compare Fig. 3c-f to Fig. 2).

## Discussion

Our results provide the first quantitative description of the dispersal rate-synchrony relationship for any ecological system. We find that even extremely low rates of dispersal, here around 0.5-1% per dispersal event (equivalent to ∼0.1–0.4% per prey generation), can produce ecologically-substantial levels of synchrony within just 2–3 cycle periods, despite starting from an initially anti-synchronous state and despite spatially-asynchronous environmental fluctuations and demographic stochasticity. It is remarkable that such low rates of dispersal can have such a strong effect on spatial population dynamics. To our knowledge, this is the first experiment in ecology to test the effects of such low rates of dispersal on spatial synchrony. Dispersal rates as low as 2.5% per event were capable of producing high synchrony. Given that interpatch movement rates in nature average approximately 15% per generation [22], our results suggest that natural dispersal rates typically will be sufficient to generate spatial synchrony, so long as natural systems are sufficiently similar to ours in other relevant respects (a caveat discussed further below).

Our results also confirm the theoretical prediction that demographic and environmental stochasticity produce substantial variation around the average behavior. Even for the highest dispersal rates used here (equivalent to >2% per prey generation), it was possible for prey metapopulations experiencing identical dispersal rates and starting from nearly-identical initial conditions to exhibit anything from near-zero synchrony to near-perfect synchrony. Inspection of the population dynamics indicates that among-replicate variability in realized synchrony was not due to sampling error, but rather reflected real variation among metapopulations and over time in the phase and period of the predator-prey cycles (i.e. in the timing and length of predator and prey "outbreaks"; Fig. 3). Just as predicted by theory (Fig. 1), our paired predator-prey populations varied greatly in how quickly they went into phase, and in whether or not they subsequently stayed in phase or drifted back out of phase (Fig. 3).

The relative roles of environmental and demographic stochasticity in generating variation in realized synchrony are unclear. Theory predicts that asynchronous environmental stochasticity can inhibit phase locking of predator-prey cycles. Fox et al. [15] found that environmental stochasticity in this system did not affect the phase of predator-prey cycles, but instead affected the synchrony of low-amplitude, environmentally-generated stochastic fluctuations in abundance superimposed on the dominant cyclic pattern. However, Fox et al. [15] noted that it was unclear if this would remain true in longer experiments, such as the one we conducted. Realized levels of synchrony were not related in any obvious way to the particular temperature time series used. Demographic stochasticity, and its desynchronizing effects, are strong when demographic events (births and deaths of individuals) are rare, as when population sizes are very low [21]. While protists are capable of achieving very large population sizes under the culture conditions used here, they can be affected by demographic stochasticity when population sizes are low, as at the nadir of a predator-prey cycle [23], [24]. That a number of our predator and prey populations went extinct illustrates that protist population sizes can drop low enough for demographic stochasticity to affect their dynamics. In future work we plan to directly test for the desynchronizing effects of demographic stochasticity by varying culture vessel size, thereby varying absolute population sizes.

Recent theory considers factors governing the rate of convergence to synchrony [25]. Our predator *Euplotes* has much slower dynamics than the prey *Tetrahymena*, leading to prey cycles that take the form of relaxation oscillations: rapid increases and subsequent crashes in prey abundance, separated by long intervals of very low density (Fig. 3). In a deterministic world, dispersal should rapidly synchronize relaxation oscillations [25]. However, long periods of low density should increase the desynchronizing effects of demographic stochasticity. Manipulating culture vessel size would allow a test for rapid convergence to synchrony at sufficiently large absolute population sizes.

Theory identifies conditions under which cycling predator-prey metapopulations will converge towards a multimodal distribution of synchrony, leading to wide variation in realized levels of synchrony among replicate metapopulations subject to both dispersal and spatially-independent demographic and environmental stochasticity [19]. It is unclear whether our system satisfies the conditions for a multimodal distribution of synchrony [19]. Testing this interesting possibility would require a much larger experiment than the one we conducted.

We were unable to detect an accelerating (concave up) relationship between synchrony and dispersal rate at very low dispersal rates, although we cannot rule it out. Possibly, upward concavity to the dispersal rate-synchrony relationship is weak or absent, or occurs only at lower dispersal rates than the ones we considered. Alternatively, among-replicate variability in the dispersal rate-synchrony relationship may have been too large for us to detect significant upward concavity at low dispersal rates.

Mean spatial synchrony reached a moderate asymptote (mean prey cross-correlation ∼0.5) at high dispersal rates, in contrast to the theoretically-predicted asymptote at near-perfect synchrony [21]. Sampling error can reduce observed synchrony by creating spatially-independent errors in population density estimates, but there are other processes contributing to this contrast. We imposed spatially-independent temperature fluctuations on our paired bottles. In previous work we showed that this reduces the prey cross-correlation in paired bottles by ∼0.3-0.4 compared to imposition of spatially-synchronized environmental fluctuations [15], [18]. In light of sampling error and spatially-independent environmental stochasticity, our demonstration of even a moderate "saturating" level of synchrony illustrates the robustness of synchrony in this experiment and ecological systems in general.

Spatial synchrony might have attained a somewhat higher asymptote had the experiment run longer, thereby allowing more time for metapopulations to achieve their asymptotic dynamics from their initially anti-synchronous state. But comparison with previous work suggests that at least our highest-dispersal metapopulations likely were relatively close to their asymptotic levels of synchrony. In previous experiments in which predator-prey cycles in different patches were started in phase or nearly in phase, and in which an intermediate dispersal rate was used, realized levels of prey synchrony under spatially-independent environmental fluctuations were either no higher than the highest levels observed in this experiment [18], or only somewhat higher [15]. Conversely, a longer experiment might not have changed the results, since given sufficient time, paired bottles can drift out of as well as into phase, a phenomenon we observed in our experiment.

We note in passing that sufficiently high rates of dispersal would produce high synchrony for the trivial reason that they would convert the metapopulation into a single well-mixed population. Our highest dispersal rate was well below the rate required to create a single well-mixed population.

Our results demonstrate that even quite low rates of dispersal, much less than 1% per generation, can generate surprisingly high spatial synchrony, even in the face of demographic and environmental stochasticity. However, demographic and environmental stochasticity can generate substantial variability in realized levels of synchrony even among ecologically-identical metapopulations. While our microcosms were not designed to mimic any particular natural system, our experiment included key factors thought to affect the synchrony of a wide range of systems. Our results suggest that, for natural species exhibiting endogenously-generated cyclic dynamics (which may be as much as 30% of populations; [9]), spatial synchrony is likely to be common. Synchrony is particularly likely in cycling systems with dispersal rates greater than a few percent per generation, since such systems likely exhibit relatively rapid convergence to synchrony.

Our experimental system can be modified to incorporate various ecological complexities, and so be used to test their effects on spatial synchrony. One major mismatch between our experiment and many natural systems is that our experiment lacked spatial environmental heterogeneity, which in nature is predicted to interfere with synchrony by causing different populations to exhibit different dynamics (e.g., cycles of different periods and amplitudes). The periods and amplitudes of microcosm predator-prey cycles can be manipulated by varying the enrichment and thickness of the culture medium and other aspects of culture conditions [26], [27]. A second potentially-important mismatch between our experiment and many natural systems is our imposition of density-independent dispersal. Under some culture conditions, some protists exhibit adaptive, density dependent dispersal when allowed to disperse themselves among microcosms interconnected by tubes [28], [29], but see [30], [31]. Theory predicts that adaptive dispersal can alter both within-patch population dynamics and their spatial synchrony [32]. Testing this theory would be an interesting direction for future work.
