# Correction: Nonlinear Effect of Dispersal Rate on Spatial Synchrony of Predator-Prey Cycles

**DOI:** 10.1371/annotation/68bea7ec-e2e6-4f8d-8ea6-ecffd145597b

**Published:** 2014-01-03

**Authors:** Jeremy W. Fox, Geoffrey Legault, David A. Vasseur, Jodie A. Einarson

The name of the second author was given incorrectly. The correct name is: Geoffrey Legault. 

